# Allergen-specific T cell quantity in blood is higher in allergic compared to nonallergic individuals

**DOI:** 10.1186/1710-1492-7-6

**Published:** 2011-04-17

**Authors:** Aito Ueno-Yamanouchi, Faisal M Khan, Bazir Serushago, Tom Bowen, Cathy Lu, Joanne Luider, Jan Storek

**Affiliations:** 1 Department of Medicine, University of Calgary, Health Science Center, 3330 Hospital Drive NW, Calgary, AB T2N 4N1, Canada; 2 Department of Pathology & Laboratory Medicine, University of Calgary, Room 269, Heritage Medical Research Building, 3330 Hospital Drive NW, Calgary, AB T2N 4N1, Canada; 3 Department of Paediatrics, University of Calgary, Room 269, Heritage Medical Research Building, 3330 Hospital Drive NW, Calgary, AB T2N 4N1, Canada

## Abstract

**Background:**

Allergen-specific IgE production is a hallmark of allergic asthma/rhinitis/eczema. Theoretically this could be due to a high number of allergen-specific B cells or allergen-specific T cells helping allergen-specific B cells differentiate into IgE-producing plasma cells. Here, we determined whether the number of allergen-specific B cells or T helper (Th) cells is higher in allergic individuals compared to nonallergic individuals.

**Methods:**

A total of 52 allergic individuals and 32 nonallergic individuals were studied. The allergen-specific B and Th cells were enumerated by culturing CFSE-loaded blood mononuclear cells for 7-days with allergen (cat, Timothy or birch), and determining the number of proliferating B or Th cells (diluting CFSE) by flow cytometry. Allergen-specific IgE concentration was determined by fluorescent enzymoimmunoassay (FEIA).

**Results:**

The quantities of proliferating Th cells but not proliferating B cells specific for cat, Timothy and birch were significantly higher in cat-, Timothy- and birch-allergic individuals compared to nonallergic individuals. The titer of allergen-specific IgE showed significant correlation with allergen-specific Th cells and not with allergen-specific B cells for all 3 allergens.

**Conclusions:**

A high number of allergen-specific proliferating Th cells, but not proliferating B cells, may play a role in the pathogenesis of allergic asthma/rhinitis/eczema.

## Background

Enhanced production of allergen-specific IgE is characteristic for allergic asthma, rhinitis or eczema [1,2]. Upon inhalation, ingestion or transcutaneous diffusion of the allergen, dendritic cells present peptides from the allergen to allergen-specific Th cells. These allergen-specific Th cells, expressing CD40 ligand and secreting Th2 cytokines like IL-4, stimulate the differentiation of allergen-specific B cells to IgE-producing plasma cells [3-6]. The increased production of IgE could be due to 1) increased quantity of allergen-specific B cells, 2) abnormal function of allergen-specific B cells (abnormally high B cell-intrinsic drive to differentiate into IgE plasma cells), 3) increased quantity of allergen-specific Th cells, 4) abnormal function of allergen-specific Th cells (abnormally high propensity to stimulate B cell differentiation into IgE plasma cells, eg, through increased secretion of Th2 cytokines), or 5) other mechanisms. To determine whether the mechanism of increased B cell quantity or the mechanism of increased Th cell quantity may apply, here we compared the quantity of allergen-specific proliferating B and Th cells for inhalant allergens in allergic and nonallergic individuals. The term allergen-specific Th cells or B cells has been used to describe allergen-specific proliferating Th or B cells throughout the manuscript. We also assessed the production of IL-4 (characteristic of Th2 cells) and IFNγ (characteristic of Th1 cells) by the allergen-specific Th cells.

## Materials and methods

### Subjects

Fifty-two allergic and 32 nonallergic individuals participated in the study. Allergic individuals were recruited by allergists (B.S. or T.B.) among patients newly referred to their allergy clinics. All 52 allergic individuals (38% male, n = 20; 62% female, n = 32) had symptoms of asthma, rhinitis or eczema and were skin prick test (SPT)-positive for at least 1 of 9 common inhalant allergens tested (see below). Their median age was 27 years (range, 18-69 years). Asymptomatic subjects (without symptoms of asthma, rhinitis or eczema) were recruited by advertising. They were included into the study as "nonallergic subjects" only if they were SPT-negative for all 9 inhalant allergens tested. We studied 32 nonallergic individuals (40% male, n = 13; 60% female, n = 19); their median age was 29 years (range, 15-47 years). During each month of blood drawing from a non allergic individual, blood was drawn also from 1-2 allergic individuals to ensure season-matching of allergic and nonallergic individuals.

To ensure uniformity in assessing the presence of symptoms of asthma, rhinitis or eczema between the symptomatic and asymptomatic persons, the International Study of Asthma and allergies in Childhood questionnaire (version Phase II, http://isaac.auckland.ac.nz/PhaseOne/Manual/ManFrame.html, accessed December 27, 2007) was used for both the symptomatic and asymptomatic subjects. Presence of symptoms was defined as a positive answer to question No. 2, 7 or 8 of the asthma section, question No. 2 of the rhinitis section or question No. 2 of the eczema section of the questionnaire. Of the 52 allergic subjects, 14 (27%) had asthma, rhinitis and eczema, 16 (31%) had asthma and rhinitis, 4 (7.5%) had rhinitis and eczema, 12 (23%) had rhinitis only, 4 (7.5%) had asthma only, and 2 (4%) had eczema only. Per another questionnaire, none of the allergic or nonallergic subjects had had cancer, autoimmune disease or immune deficiency, had ever received allergen immunotherapy or received systemic immunosuppressive drugs in the previous three months. None of the subjects received antihistamines in the last 7 days prior to SPT. All subjects (allergics and nonallergics) signed a written consent to participate in the study. The study was approved by the Ethics Committee of the University of Calgary.

Blood was drawn for allergen-specific B/Th cell assays prior to SPT (typically within one hour prior to SPT) to eliminate the possibility of SPT influence on the results of the allergen-specific B/Th cell assays. Blood was drawn at two different times from 5 allergic and 4 nonallergic individuals to evaluate whether the quantity of allergen specific B and Th cells differs in the same individual at different time points.

### Allergens

Allergen extracts (ALK-Abello, Horsholm, Denmark, except for Timothy grass pollen extract from Greer Laboratories, Lenoir, NC, USA) were kindly donated by Western Allergy, Mississauga, Ontario, Canada. Neat extracts contained 50% glycerol and 0.4% phenol. Negative control was 0.9% sodium chloride in 50% glycerol and 0.4% phenol (Glycerol Saline). Positive control was histamine 1 mg/mL in 50% glycerol and 0.4% phenol (Histatrol, [ALK-Abello, Horsholm, Denmark]) for skin prick test and monoclonal mouse-anti-human CD3 (mitogenic clone 64.1) for allergen-specific Th cells assay. The same CD3 antibody was used also as a positive control for the allergen-specific B cell assay, as B cell proliferation was induced in the CD3 antibody-stimulated culture of mononuclear cells, probably by stimulated T cells. The allergen concentration used for SPT was in compliance with the US guidelines on probable effective concentration range for allergen extracts (http://www.aaaai.org/professionals/resources/immunotherapy/, accessed on November 26, 2008). The allergen concentration used for allergen-specific B/Th cells assay was based on our preliminary experiments in which assay was performed for each allergen using three different concentrations - 10-times, 100-times and 1000-times lower concentration than that used for SPT. The 100-times lower concentration was associated with the highest percentage of Th and B cell proliferation above Glycerol Saline background. Thus, the final concentration used was as follows:

○ Cat pelt, 10,000 BAU/ml [SPT], 100 BAU/ml [specific B/Th cells]

○ Dog epithelium, 1:20 [SPT], 1:2000 [specific B/Th cells]

○ *Dermatophagoides pteronyssius (DP*), 10000 AU/ml [SPT], 100 AU/ml [specific B/Th cells]

○ *Dermatophagoides farinae (DF)*, 10000 AU/ml [SPT], 100 AU/ml [specific B/Th cells]

○ Alternaria, 1:10 [SPT], 1:1000 [specific B/Th cells]

○ Hormodendrum/Cladosporium, 1:10 [SPT], 1:1000 [specific B/Th cells]

○ Timothy grass pollen, 100,000 AU/ml [SPT], 1000 AU/ml [specific B/Th cells]

○ Short ragweed pollen, 1:20 [SPT], 1:2000 [specific B/Th cells]

○ Birch tree (*Betula verrucosa*) pollen, 1:20 [SPT], 1:2000 [specific B/Th cells]

### Enumeration of Allergen-Specific B, Th, Th1 and Th2 cells

#### (a) Cell culture and Flow analysis

**Figure 1:**
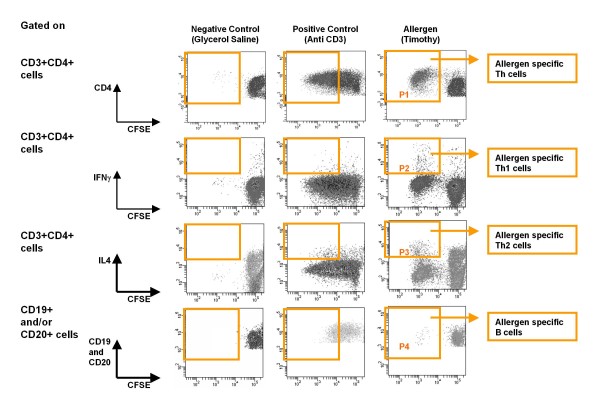
**Example of allergen specific Th and B cells**. Peripheral blood MNCs (in this example from Timothy-allergic individual per skin prick test) labeled with carboxyfluorescein diacetate succinimidyl ester (CFSE) were cultured for 7 days with Timothy allergen. Glycerol saline (negative control) and anti-CD3 (positive control) were used as controls. Monensin was used to stop cytokine secretion. At the end of the culture, cells were stained for CD3, CD4, CD19, CD20, IFNg and IL-4 and analyzed using FACS Aria flow cytometer. Timothy-specific Th, Th1, Th2 and B cells are defined as CFSE^low^CD3^pos^CD4^pos ^(P1), CFSE^low^CD3^pos^CD4^pos^IFNg^pos ^(P2), CFSE^low^CD3^pos^CD4^pos ^IL-4 ^pos ^(P3) and CFSE^low^CD3^pos^(CD19 ^pos ^or CD20 ^pos^) (P4) cells, respectively.

Blood was drawn into heparinized tubes. Within 8 h from the blood draw, mononuclear cells (MNCs) were isolated using density gradient centrifugation (Ficoll, density 1.073 kg/L) and labeled with 5 μM carboxyfluorescein diacetate succinimidyl ester (CFSE, Molecular Probes). CFSE labeling was done to measure the proliferation of allergen-specific Th and B cells. When a CFSE-labeled cell divides, CFSE-labeled proteins in the cell are equally distributed between the daughter cells, thus halving cell fluorescence with each division. Consequently, dividing cells lose their fluorescence (become CFSE^low^), and non-proliferating cells preserve their brightness (remain CFSE^high^). The number of the original cells can be calculated from estimated number of divisions for each cell [7]. This allows the detection of low frequency cells that can only be detected after they have proliferated. Three million of CFSE-labeled MNCs in 2 ml of DMEM-RS media (Hyclone, Logan, UT) supplemented with 2 mM glutamine, Penicillin (100 U/ml), Streptomycin (0.1 mg/ml), and 5% autologous plasma were incubated with allergen (see "Allergens", above, for concentration) or negative control (Glycerol Saline) or positive control (anti-CD3) for 7 days at 37°C in a humidified atmosphere containing 5% CO_2_. Monensin (Golgistop, BD Biosciences; final concentration 2 mM) was added into the cell culture on day 6 (for the last 18 h of culture). At the end of culture, cells were washed using PBS with 10% Fetal Bovine Serum and 2 mM EDTA, resuspended in PBS, and fixed and permeabilized using BD cytofix/cytoperm kit (BD Biosciences). Then the cells were stained for 30 min at 4°C with the following fluorochrome-labeled antibodies: IFNγ-APC, CD4-APC-Cy7 (Miltenyi Biotec, Bergisch Gladbach, Germany), IL-4-PE, CD3-PC7 (BD Biosciences, San Jose, CA, USA) and CD19-PC5 and CD20-PC5 (Beckman Coulter, Mississauga, Ontario, Canada). Cells were washed and resuspended in PBS with 1% bovine serum albumin and 0.1% sodium azide. Immediately before flow cytometry, a known number of fluorospheres (eg, 50,000) (Flow-Count, Beckman Coulter) were added to each sample. The cells were then analyzed by flow cytometry (FACS Aria, BD Biosciences, San Jose, CA, USA). Data were analyzed using FACS DiVa software (BD Biosciences, San Jose, CA, USA). Allergen-specific B cells were defined as CFSE^low ^cells expressing CD19 or CD20. Allergen-specific Th cells were defined as CFSE^low ^cells expressing CD3 and CD4 (Figure 1). Allergen-specific Th1 or Th2 cells were defined as allergen-specific CFSE^low ^cells expressing CD3 and CD4 and either IFNg (Th1) or IL-4 (Th2) (Figure 1).

#### (b) Index and absolute count of allergen specific B, Th, Th1 and Th2 cells

**Figure 2:**
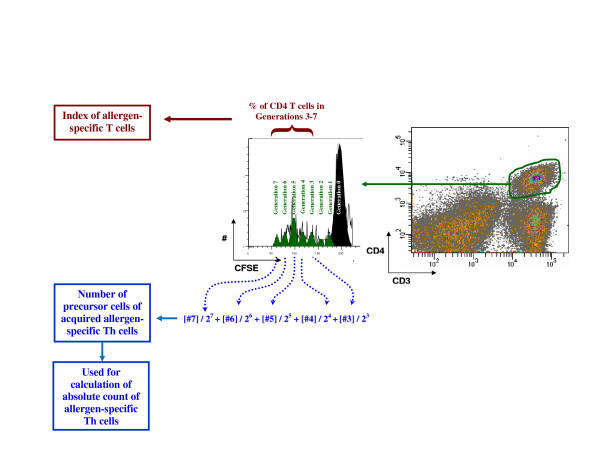
**Example of calculation of the index of the quantity of allergen-specific Th cells and the number of precursor cells of acquired allergen-specific cells (needed to calculate the absolute count of allergen specific Th cells)**. The percentage of the CFSE^low ^cells after the 7 day culture is referred to as the "index" of the quantity of allergen-specific cells (in this example, Timothy-specific Th index). The number of precursor cells of acquired allergen-specific cells (in this example, precursor cells of CFSE^low ^Th cells), determined using the ModFit software, and was used to estimate the absolute count of allergen-specific Th cells. Only generations 3 and higher were considered as allergen-specific cells and generations 0, 1 and 2 were omitted from the calculation.

The method of calculation of index and absolute count of allergen specific B, Th, Th1 and Th2 cells is displayed in Figure 2. The percentage of the CFSE^low ^cells on day 7 of culture is referred to as the "index" of the quantity of allergen-specific cells. The absolute count of the allergen-specific cells was determined from the absolute MNC count on day 0 (absolute lymphocyte count + absolute monocyte count per ml of blood), the acquired cell proportion on day 7 (determined as the acquired proportion of fluorospheres, eg, 0.8 if 40,000 of the 50,000 fluorospheres were acquired), and the number of precursor cells of acquired (by flow cytometry) allergen-specific cells (determined using Modfit software, Verity Software House, see next paragraph for details). The absolute count of allergen-specific cells (per ml of blood) was calculated using the following formula:

*B cells, Th cells, Th1 cells or Th2 cells

The number of precursor cells of acquired allergen-specific cells (the precursor cells of CFSE^low ^Th cells, CFSE^low ^B cells, CFSE^low ^IFNγ+ Th cells, CFSE^low ^IL-4+ Th cells) was estimated using the ModFit software (Verity Software House, Topsham, ME, USA). Based on CFSE fluorescence, the software estimates how many cells divided (between day 0 and day 7) once (generation 1), twice (generation 2), three times (generation 3), etc. To exclude bystander responding cells (which should undergo fewer divisions than allergen-specific cells), only generations 3, 4, 5 and higher were considered as the allergen-specific cells and generations 0, 1 and 2 were omitted from the calculation (Figure 2). The number of precursor cells of acquired allergen-specific cells was calculated as ([number of cells in generation 3]/2^3 ^+ [number of cells in generation 4]/2^4 ^+ [number of cells in generation 5]/2^5 ^+ [number of cells in generation 6]/2^6 ^+ [number of cells in generation 7]/2^7 ^+ [number of cells in generation 8]/2^8^).

**Figure 3:**
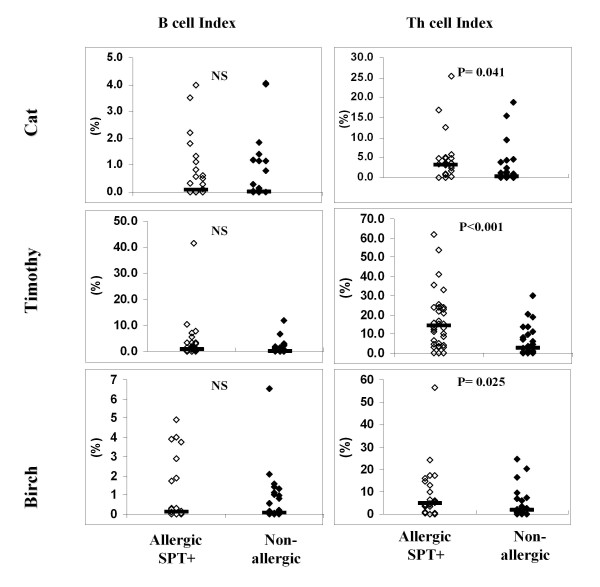
**Indices for allergen-specific B cells *(left) *and Th cells *(right) *in allergic patients (n = 52, closed diamonds) and nonallergic persons (n = 32, open diamonds)**. The numbers of allergic patients were 26 for cat, 34 for Timothy, and 27 for birch. Significance of the difference between the allergic and nonallergic groups is given in the upper section of each plot. Allergen-specific Th and B cell results are displayed as corrected percentage of CFSE low Th and B cells (saline control percentage subtracted). The horizontal bars show the medians.

**Figure 4:**
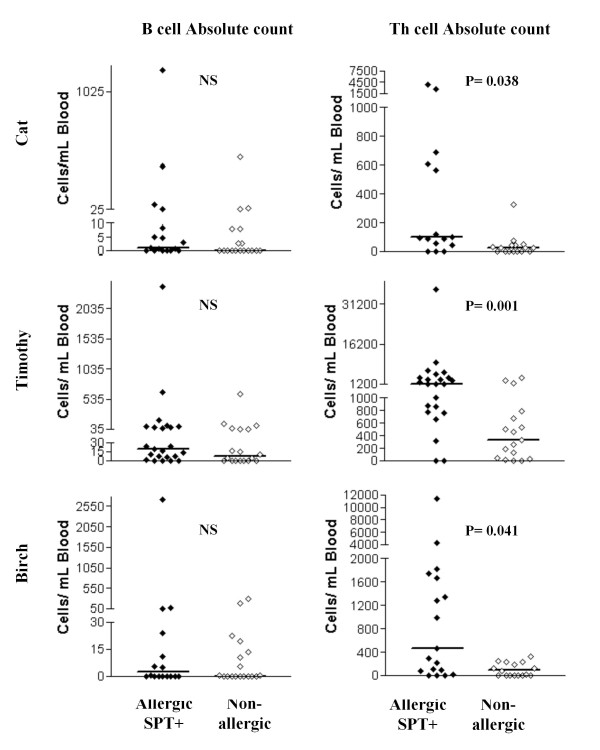
**Absolute count for allergen-specific B cells *(left) *and Th cells *(right) *in allergic patients (n = 33, closed diamonds) and nonallergic persons (n = 18, open diamonds)**. The numbers of allergic patients were 18 for cat, 23 for Timothy, and 19 for birch. Significance of the difference between the allergic and nonallergic groups is given in the upper section of each plot. Allergen-specific Th and B cells results are displayed as corrected absolute count of CFSE low Th and B cells (saline control absolute counts subtracted). The horizontal bars show the medians.

To correct the index or the absolute count of allergen-specific cells for background (eg, due to nonspecific stimulation, nonspecific staining or loss of CFSE activity), the index or the absolute count of the negative control was subtracted. The indices and absolute counts presented in the Results and Figures 3 and 4 are the corrected indices and corrected absolute counts.

### Skin Prick Testing

Allergen drops and positive and negative control drops were applied on the volar forearms with at least 2 cm distance from each other. For each allergen, a single epicutaneous prick was done using Allersharp^® ^device (Western Allergy, Mississauga, Ontario, Canada). Wheal area was recorded for Histatrol at 10 min, and for others (each allergen and negative control) at 15 min by outlining the area with a felt-tipped pen, and transferring the outline onto 3 M tapes to keep a permanent record of SPT. The recorded wheal areas were scanned as jpeg files and analyzed by Image J software (National Institutes of Health, Bethesda, MD, USA) to determine the average diameter of each wheal. The average diameter of the negative control wheal was subtracted from each allergen wheal (corrected diameter). The SPT result was considered positive if the corrected diameter was greater than 3 mm [8]. All subjects had a valid SPT as defined by at least 1 mm diameter difference between the positive and negative controls[8].

### Fluorescent enzymoimmunoassay

Sera from the research subjects were stored in tightly sealed vials at -86°C. Allergen-specific IgE concentration was determined using UniCAP100 instrument and specific IgE FEIA reagents (Phadia, Uppsala, Sweden, accessed January 7, 2010) per manufacturer instructions. Numbers of allergic and non-allergic individuals tested for allergen-specific IgE are mentioned in Table 1.

**Table 1:** Subjects (allergic and nonallergic) studied

Allergens	Index Analysis		Absolute count Analysis		FEIA analysis	
	**Allergic Individuals**^**1**^	**Nonallergic Individuals**^**2**^	**Allergic Individuals**^**1**^	**Nonallergic Individuals**^**2**^	**Allergic Individuals**^**1**^	**Nonallergic Individuals**^**2**^

**Cat**	26	32	18	18	13	12
**Timothy**	34	32	23	18	11	12
**Birch**	27	32	19	18	12	12

^1 ^Numbers denote the number of allergic individuals (with symptoms of asthma/rhinitis/eczema and skin prick test (SPT) positive for the specific allergen)

^2 ^Numbers denote the number of nonallergic individuals (with no symptoms of asthma/rhinitis/eczema and SPT negative for all 9 allergens tested)

### Statistics

Significance of difference of test results (index or the absolute count of allergen-specific T or B cells) between 2 subject groups was tested by Mann-Whitney-Wilcoxon rank sum test. P values less than 0.05 (2-tailed) were considered significant.

## Results

### Allergic and nonallergic individuals

By SPT, 26 (50%) of the 52 allergic subjects were allergic to cat, 14 (27%) to dog, 11 (21%) to *D. pteronyssimus*, 6 (11%) to *D. farinae*, 2 (4%) to Alternaria, 3 (6%) to Hormodendrum, 34 (65%) to Timothy, 27 (52%) to birch and 6 (11%) to short ragweed. Given the small number of subjects allergic to *Dog, D. pteronyssimus, D. farinae*, Alternaria, Hormodendrum and short ragweed, only analyses pertinent to cat, Timothy and birch are presented here. The indices of allergen-specific T/B cells were determined in all 52 allergic and 32 nonallergic individuals, whereas the absolute counts were determined in only the last consecutive 33 allergic and 18 nonallergic individuals (Table 1).

### Indices of allergen-specific B and Th cells

Indices of allergen-specific B cells were similar in individuals allergic to any of the allergens analyzed (cat, Timothy, birch) compared to nonallergic individuals (Figure 3, *left*). In contrast, the indices of allergen-specific Th cells were significantly higher in individuals allergic to cat, Timothy or birch compared to nonallergic individuals (Figure 3, *right*).

### Absolute counts of allergen-specific B and Th cells

The indices presented in the previous paragraph are imperfect indicators of the quantity of allergen-specific B/Th cells. For example, a higher allergen-specific Th cell index in allergic individuals could be due to the fact that allergen-specific Th cells from allergic individuals underwent on average more divisions in the culture than allergen-specific Th cells from nonallergic individuals. The index also does not take into account potential differences in the absolute counts of total B or Th cells in the blood of allergic and nonallergic individuals. Thus, in a subset of the study subjects (the "n" for each allergen is given in Table 1) we also determined the absolute counts of allergen-specific B and Th cells.

**Figure 5:**
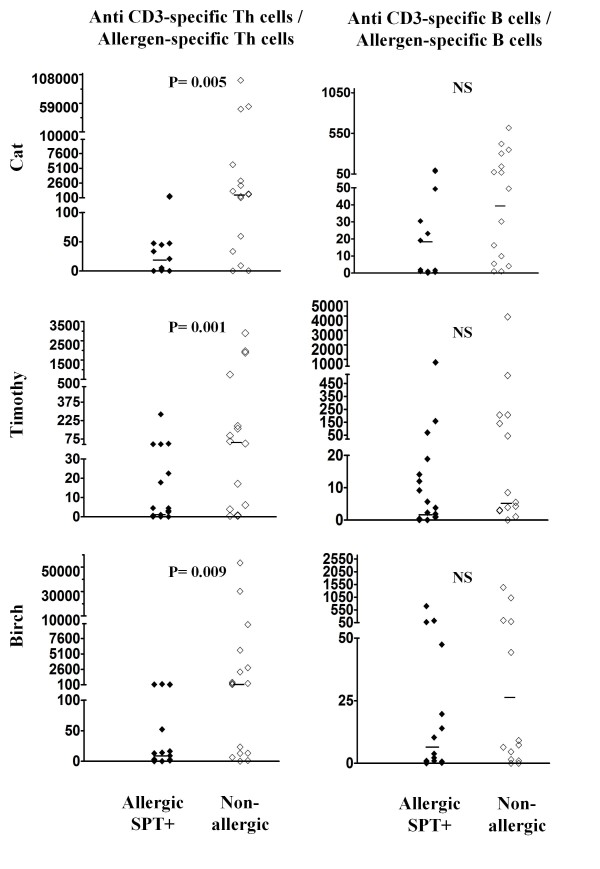
**Ratio of positive control (anti CD3)-specific Th cell and allergen-specific Th cells *(left) *and ratio of positive control (anti CD3)-specific B cells and allergen-specific B cells *(right) *in allergic patients *(n = 33, closed diamonds) *and nonallergic persons *(n = 18, open diamonds)***. The numbers of allergic patients were 18 for cat, 23 for Timothy, and 19 for birch. Significance of the difference between the allergic and nonallergic groups is given in the upper section of each plot. The horizontal bars show the medians.

Analogous to the indices, the absolute counts of allergen-specific B cells were similar in individuals allergic to any of the allergens analyzed compared to nonallergic individuals (Figure 4, *left*). Also analogous to the indices, the absolute counts of allergen-specific Th cells were significantly higher in individuals allergic to cat, Timothy or birch compared to nonallergic individuals (Figure 4, *right*). We then compared the ratio of positive control (anti CD3)-specific Th cell and allergen-specific Th cells to rule out the impact of run variability. Similar to the absolute counts of allergen-specific Th cells, the ratio of positive control (anti CD3)-specific Th cell and allergen-specific Th cells was significantly higher in individuals allergic to cat, Timothy or birch compared to nonallergic individuals (Figure 5). The absolute counts of Th and B cells were found similar in allergic individuals allergic to one and more than one allergen.

In order to assess whether the observed proliferation of Th cells was allergen-specific, we performed an additional analysis by dividing allergic patients into those allergic to the allergen of interest (SPT result positive for that allergen, "Allergic SPT+") and those allergic to a different allergen(s) ("Allergic SPT-"). For example, cat allergen acts as a non-offending allergen in CAT SPT- allergic individuals. The absolute counts of allergen-specific Th cells were higher for all 3 allergens tested in SPT+ than SPT- individuals (Additional file 1, Figure S1). The difference was significant for Timothy (P = 0.001) and showed a trend of significance for birch (P = 0.06) and cat (P = 0.09). Also, the absolute counts of Th cells specific for Timothy, birch and cat were similar in SPT- and non-allergic individuals. In contrast to T cells, absolute counts of B cells specific for Timothy, birch and cat were similar between SPT+ and SPT-individuals, SPT+ and non-allergic individuals and SPT- and non-allergic individuals (Additional file 1, Figure S1).

### Correlation between allergen-specific IgE and allergen-specific Th and B cells

Since production of allergen-specific IgE is characteristic for allergic diseases, we determined serum concentration of allergen-specific IgE using FEIA in 22 allergic and 12 non-allergic individuals. As expected, the absolute counts of allergen-specific IgE were significantly higher in individuals allergic to cat, Timothy or birch compared to nonallergic individuals (Additional file 2, Figure S2). Correlations between allergen-specific IgE and allergen-specific Th cells but not allergen-specific B cells (specific for cat, Timothy and birch) were statistically significant (Table 2).

**Table 2:** Correlation between allergen-specific IgE obtained from FEIA and absolute count of allergen-specific Th and B cells.

	Allergen-specific IgE vs Allergen-specific Th cells	Allergen-specific IgE vs Allergen-specific B cells
**Cat**	R = .58	R = .39
	P = .004	P = .07
**Timothy**	R = .72	R = .38
	P <.001	P=.08
**Birch**	R = .50	R = .21
	P = .01	P = 76

### Indices and absolute counts of allergen-specific Th1 and Th2 cells

No significant differences in the indices or absolute counts of allergen-specific Th1 or Th2 cells between the individuals allergic to any of the allergens analyzed and nonallergic individuals were observed. There were trends toward higher indices and absolute counts of both allergen-specific Th1 cells and allergen-specific Th2 cells in allergic compared to nonallergic individuals; statistical significance was not reached probably due to a high interindividual variabily in the number of allergen-specific Th1 as well as Th2 cells.

### Intraindividual variability of allergen-specific Th and B cells (in blood drawn on different dates) is remarkably low

**Figure 6:**
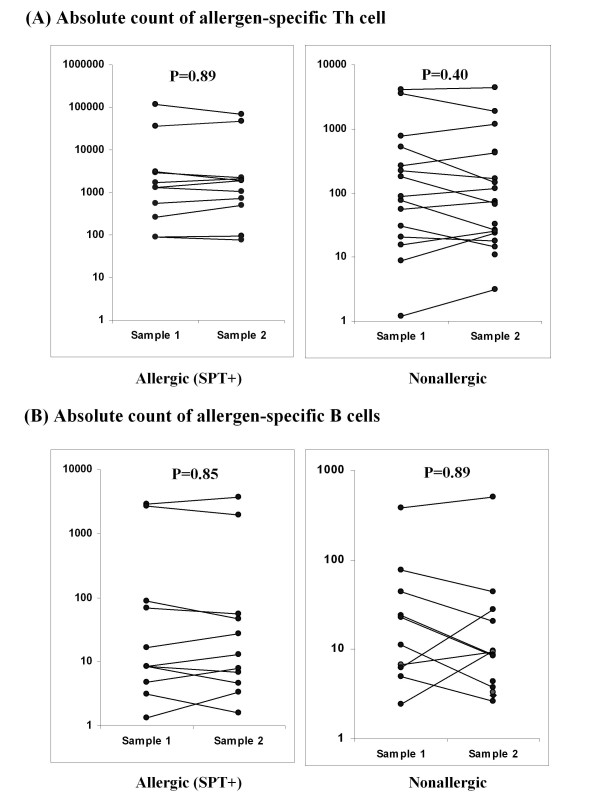
**Comparison of absolute count of (A) allergen-specific Th and (B) allergen-specific B cells in blood drawn at different time point from 5 allergic and 4 nonallergic individuals**. Significance of the difference between samples (1 and 2) collected from same individual is given in the upper section of each plot. If blood from a patient was drawn on more than two occasions, sample 3 was considered as sample 1 or 2 for each of the other 2 samples. Allergen-specific Th and B cell results are displayed as corrected absolute count of CFSE low Th and B cells (saline control absolute counts subtracted).

In spite of the statistically significant difference in cat/Timothy/birch-specific Th cell counts between allergic and nonallergic individuals, there were cat/Timothy/birch-allergic individuals with low cat/Timothy/birch-specific Th cell counts (in the range of nonallergic individuals) as well as nonallergic individuals with high cat/Timothy/birch-specfic Th cell counts (in the range of allergic individuals) (Figure 6). This could be either due to a high variability in assay results (due, eg, to variation of allergen-specific Th cell counts from month to month [eg, due to season or technical reasons] or because allergen-specific Th cells were truly low in some allergic individuals or truly high in some nonallergic individuals. Also, the lack of statistically significant difference in allergen-specific B cell counts between allergic and nonallergic individuals could be due to a high variability in assay results. Thus, we drew blood from 5 cat/Timothy/birch-allergic individuals and 4 nonallergic individuals at ≥2 time points, and measured allergen-specific Th and B cells at each time point. As shown in Figure 5, the results were remarkably similar between time points. This implies that (1) cat/Timothy/birch-specific Th cell counts are high in most but not all cat/Timothy/birch-allergic individuals and low in most but not all nonallergic individuals, and (2) the lack of statistically significant difference between allergen-specific B cell counts in allergic vs nonallergic individuals is likely not due to a high variability of the assay results.

## Discussion

The present study shows three important findings. The foremost among them is the similarity of allergen-specific proliferating B cell quantity (index or absolute count) in allergic and nonallergic individuals. Irsch et al. and Burastero et al. have also described similar frequency of allergen-specific B cells in allergic vs nonallergic individuals; however absolute counts have not been determined in those studies [9,10]. Our data together with those of Irsch et al. and Burastero et al. suggest that since the quantity of allergen-specific B cells in blood is not different between allergic and non-allergic individuals, other mechanisms like increased differentiation of allergen-specific B cells into IgE-producing plasma cells may be involved in the pathogenesis of allergic diseases. This notion is further supported by the poor correlation of allergen-specific B cell counts with serum levels of allergen-specific IgE. Consistent with this speculation, Kasaian et. al showed that allergic individuals have higher frequencies of IgE-producing B cells in peripheral blood than nonallergic individuals [6,7]. These are likely the precursors of IgE-producing plasma cells that are increased in number in the airways of allergic individuals [11].

The second crucial finding of this study is that the quantity of cat, Timothy and birch-specific Th cells is higher in allergic than nonallergic individuals and significantly correlates with serum levels of cat, Timothy and birch-specific IgE. We did not find a significant difference in the Th or B cell indices or absolute counts between individuals allergic to other allergens (eg, dog, DP) but this may be due to the inadequate power to detect a difference (data not shown). Specificity of allergen-specific activation of Th cells was demonstrated by showing that allergen-specific Th cells for all three allergens (cat, Timothy, birch) was consistently higher in allergic SPT+ than SPT- individuals and similar between allergic SPT- and non-allergic individuals. In previous studies allergen-specific Th cell quantities were also suspected to be increased (based on increased thymidine incorporation by MNCs or increased frequencies of proliferating T cells stimulated with allergen) in persons allergic to that allergen compared to nonallergic persons in case of some allergens (eg, cow's milk protein, peanut)[12-14] but not other allergens (eg, ovalbumin) [14]. However, absolute counts were not determined. In our study, both indices and absolute counts for allergen specific Th cells have shown a similar pattern (significantly different for cat, Timothy and birch). Consistent with our results, Tay et al showed a higher frequency of peanut-specific T cells in peanut-allergic individuals compared to controls but similar frequency of egg allergen-specific T cells in egg-allergic compared to nonallergic subjects [14]. We speculate that in allergic persons, the increased number of allergen-specific Th cells may drive the allergen-specific B cells to differentiate into IgE plasma cells. This may have been the reason why allergen-specific Th cells showed consistently better correlation with serum titer of allergen-specific IgE than allergen-specific B cells.

The third important finding of the study is the similarity in allergen-specific Th cell quantity when analyzed at different time-points. This suggests that the interassay variability is low and that the quantity of allergen specific Th cells remains relatively constant irrespective of season or allergen exposure. More important, this suggests that despite cat/Timothy/birch-allergic individuals have higher-than-normal allergen-specific Th cell counts as a group, there are some cat/Timothy/birch-allergic patients with low allergen-specific T cell counts and some nonallergic patients with high allergen-specific T cell counts. This suggests that the high allergen-specific Th cell count is not the only pathogenic mechanism of allergic disease, as other mechanisms may cause an individual with low allergen-specific Th cell counts to develop allergic disease or an individual with high allergen-specific Th cell counts not to develop allergic disease.

There are important limitations of the interpretation of our findings of no difference in the quantity of allergen-specific B cells between allergic and nonallergic individuals in case of all allergens tested. First, we enumerated the allergen-specific B or Th cells in blood and not in bone marrow, lymphoid and non-lymphoid tissues like skin or mucosas. The non-blood tissues might house the majority of allergen-specific memory lymphocytes. So it is theoretically possible that allergic individuals have higher numbers of allergen-specific B cells in tissues like the airway mucosa or lymphoid organs like tonsils. Though, it is important to note in this regard that the numbers of total lymphocytes, B cells and CD4 T cells in airway secretions or biopsy specimens have been shown to be similar between allergic and nonallergic individuals[15]. The second limitation is that enumeration of allergen-specific B cells is based on their proliferation. Other functions like production of IgE have not been studied. The third limitation is that our method of enumeration of the allergen-specific Th or B cells was based on the ability of the cells to proliferate. We assumed that all allergen-specific cells proliferated and that their death rate during the 7 days of culture was negligible. Fourth, for B cells, using a positive control stimulus directly stimulating B cells like CD40L might have been advantageous. Fifth, we did not take into account cell loss during cell staining (including washes), as we considered this to be negligible and likely similar between allergic and nonallergic individuals. The correctness of these assumptions has not been tested.

Increased Th2 cell or decreased Th1 cell quantity or function has been considered to play a role in the pathogenesis of allergic disease [13,16-18] though this has been recently contested [14]. The production of IL-4 by IL-4-secreting Th cells (judged by anti-IL-4-PE fluorescence of IL-4^+ ^cells) did not appear to be higher in our allergic than nonallergic subjects, and the production of IFNγ by IFNγ-secreting Th cells also did not appear to be lower. Tay et al has also shown that there were no significant differences in IFN-γ or IL-4 producing cell numbers or in IFN-γ/IL-4 ratios between peanut- and egg- allergic and nonallergic groups [14]. This suggests against a pathogenic role of increased Th2 or decreased Th1 function. However, the role of Th1 and Th2 cells cannot be conclusively determined from our study, as the secretion of IL-4 and IFNγ was measured after the cells were cultured for 6-7 days - during this period their ability to secrete IL-4 or IFNγ may have changed. It is also important that only some, but not all T cell clones and lines specific for allergens that were cultured from peripheral blood of allergic subjects have been shown to display Th2 phenotypes [19-21].

## Conclusions

The increased production of allergen-specific IgE in patients with allergic asthma, rhinitis or eczema probably does not result from an increased number of allergen-specific B cells but might result from an increased number of allergen-specific Th cells that could stimulate differentiation of allergen-specific B cells into IgE-producing plasma cells.

## Competing interests

The authors declare that they have no competing interests.

## Authors' contributions

AUY and FMK performed experiments. AUY performed analysis and generated the initial draft of the manuscript, FMK performed experiments, carried out final analyses and wrote later drafts of the manuscript, BS and TB recruited allergic subjects and performed skin prick tests, CL was involved in performing laboratory experiments assay, JL was involved in data analyses and JS designed the study, edited the drafts and the final version of the manuscript. FMK is Barb Ibbotson ACHF Investigator in Pediatric Hematology. JS is a recipient of Canada Research Chair and Alberta Heritage Foundation Clinical Scholar Awards. All authors have critically reviewed, and approved the final manuscript.

## Supplementary Material

Additional file 1 **Figure S1: Absolute count for allergen-specific B cells *(left) *and Th cells *(right) *in allergic SPT + patients (closed diamonds), nonallergic persons (open diamonds), and allergic SPT - patients (open circle)**. The allergic patients are divided into those allergic to the allergen of interest per SPT result ("Allergic SPT+") and those allergic to a different allergen(s) ("Allergic SPT-"). The numbers of Allergic SPT+ patients were 18 for cat, 23 for Timothy and 19 for birch. The numbers of Allergic SPT- patients can be calculated for each allergen as 39 minus the number of Allergic SPT+ patients (eg, 33-23 = 10 for cat). Significance of the difference between the Allergic SPT+ and Allergic SPT- groups and between Allergic SPT- and Nonallergic groups is given in the upper section of each plot. The horizontal bars show the medians. Click here for file

Additional file 2 **Figure S2: Allergen-specific IgE values analyzed by FEIA in allergic (n = 22, closed diamonds) and nonallergic (n = 12, open diamonds) individuals**. The numbers of allergic individuals were 13 for cat, 11 for Timothy, and 12 for birch. Significance of the difference between the allergic and nonallergic individuals is given in the upper section of each plot. Undetectable IgE levels by FEIA are displayed as 0.05 kU/L. The horizontal bars show the medians. Click here for file
