# Supplementary material for: Allergen-specific T cell quantity in blood is higher in allergic compared to nonallergic individuals
Source: Allergy Asthma Clin Immunol. 2011 Apr 17;7(1):6. doi: 10.1186/1710-1492-7-6 (PMC3102632; doi:10.1186/1710-1492-7-6)
Supplement: Additional file 1 — Figure S1: Absolute count for allergen-specific B cells (left) and Th cells (right) in allergic SPT + patients (closed diamonds), nonallergic persons (open diamonds), and allergic SPT - patients (open circle). The allergic patients are divided into those allergic to the allergen of interest per SPT result ("Allergic SPT+") and those allergic to a different allergen(s) ("Allergic SPT-"). The numbers of Allergic SPT+ patients were 18 for cat, 23 for Timothy and 19 for birch. The numbers of Allergic SPT- patients can be calculated for each allergen as 39 minus the number of Allergic SPT+ patients (eg, 33-23 = 10 for cat). Significance of the difference between the Allergic SPT+ and Allergic SPT- groups and between Allergic SPT- and Nonallergic groups is given in the upper section of each plot. The horizontal bars show the medians. [file 1710-1492-7-6-S1.PDF]

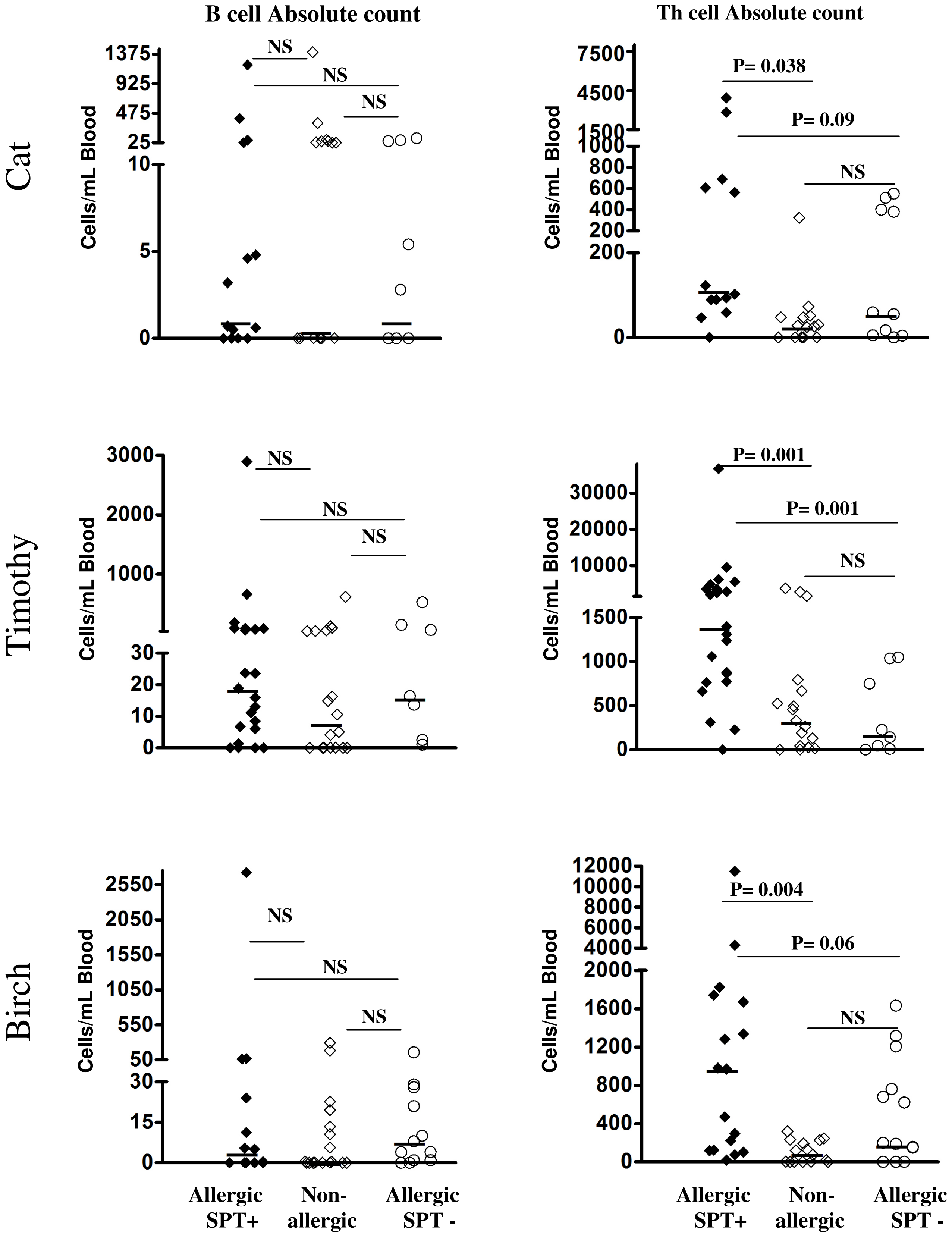

B cell Absolute count
Th cell Absolute count
Cat
Timothy
Birch
Cells/mL Blood
NS
P= 0.038
P= 0.09
P= 0.001
P= 0.001
P= 0.004
P= 0.06
Allergic SPT+
Non-allergic
Allergic SPT -

Additional File1, Figure S1

Title: Absolute count for allergen-specific B cells *(left)* and Th cells *(right)* in allergic SPT + patients (closed diamonds), nonallergic persons (open diamonds), and allergic SPT – patients (open circle).

The allergic patients are divided into those allergic to the allergen of interest per SPT result ("Allergic SPT+") and those allergic to a different allergen(s) ("Allergic SPT-"). The numbers of Allergic SPT+ patients were 18 for cat, 23 for Timothy and 19 for birch. The numbers of Allergic SPT- patients can be calculated for each allergen as 39 minus the number of Allergic SPT+ patients (eg, 33-23=10 for cat). Significance of the difference between the Allergic SPT+ and Allergic SPT- groups and between Allergic SPT- and Nonallergic groups is given in the upper section of each plot. The horizontal bars show the medians.
