# Supplementary material for: Allergen-specific T cell quantity in blood is higher in allergic compared to nonallergic individuals
Source: Allergy Asthma Clin Immunol. 2011 Apr 17;7(1):6. doi: 10.1186/1710-1492-7-6 (PMC3102632; doi:10.1186/1710-1492-7-6)
Supplement: Additional file 2 — Figure S2: Allergen-specific IgE values analyzed by FEIA in allergic (n = 22, closed diamonds) and nonallergic (n = 12, open diamonds) individuals. The numbers of allergic individuals were 13 for cat, 11 for Timothy, and 12 for birch. Significance of the difference between the allergic and nonallergic individuals is given in the upper section of each plot. Undetectable IgE levels by FEIA are displayed as 0.05 kU/L. The horizontal bars show the medians. [file 1710-1492-7-6-S2.PDF]

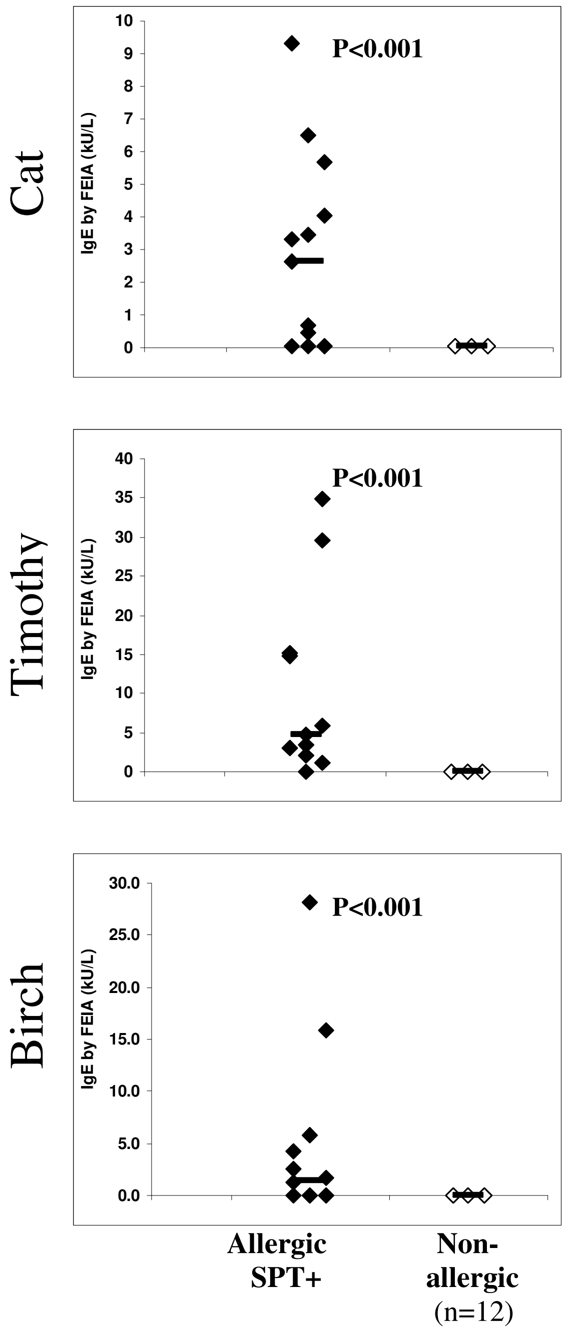

Additional File 2, Figure S2

Title: Allergen-specific IgE values analyzed by FEIA in allergic (n=22, closed diamonds) and nonallergic (n=12, open diamonds) individuals.

The numbers of allergic individuals were 13 for cat, 11 for Timothy, and 12 for birch. Significance of the difference between the allergic and nonallergic individuals is given in the upper section of each plot. Undetectable IgE levels by FEIA are displayed as 0.05 kU/L. The horizontal bars show the medians
